# Effects of Forearm Compression Sleeves on Muscle Hemodynamics and Muscular Strength and Endurance Parameters in Sports Climbing: A Randomized, Controlled Crossover Trial

**DOI:** 10.3389/fphys.2022.888860

**Published:** 2022-06-03

**Authors:** Mirjam Limmer, Markus de Marées, Ralf Roth

**Affiliations:** ^1^ Institute of Outdoor Sports and Environmental Science, German Sports University Cologne, Cologne, Germany; ^2^ Department of Sports Medicine and Sports Nutrition, Faculty of Sport Science, Ruhr-University Bochum, Bochum, Germany

**Keywords:** sport climbing, muscle oxygenation, near infrared spectroscopy, compression garments, hand grip strength, finger flexor muscles

## Abstract

**Purpose:** Wearing compression garments is a commonly used intervention in sports to improve performance and facilitate recovery. Some evidence supports the use of forearm compression to improve muscle tissue oxygenation and enhance sports climbing performance. However, evidence is lacking for an effect of compression garments on hand grip strength and specific sports climbing performance. The purpose of this study was to evaluate the immediate effects of forearm compression sleeves on muscular strength and endurance of finger flexor muscles in sports climbers.

**Materials and Methods:** This randomized crossover study included 24 sports climbers who performed one familiarization trial and three subsequent test trials while wearing compression forearm sleeves (COMP), non-compressive placebo forearm sleeves (PLAC), or no forearm sleeves (CON). Test trials consisted of three performance measurements (intermittent hand grip strength and endurance measurements, finger hang, and lap climbing) at intervals of at least 48 h in a randomized order. Muscle oxygenation during hand grip and finger hang measurements was assessed by near-infrared spectroscopy. The maximum blood lactate level, rate of perceived exertion, and forearm muscle pain were also determined directly after the lap climbing trials.

**Results:** COMP resulted in higher changes in oxy[heme] and tissue oxygen saturation (StO_2_) during the deoxygenation (oxy[heme]: COMP –10.7 ± 5.4, PLAC –6.7 ± 4.3, CON –6.9 ± 5.0 [μmol]; *p* = 0.014, η_p_^2^ = 0.263; StO_2_: COMP –4.0 ± 2.2, PLAC –3.0 ± 1.4, CON –2.8 ± 1.8 [%]; *p* = 0.049, η_p_^2^ = 0.194) and reoxygenation (oxy [heme]: COMP 10.2 ± 5.3, PLAC 6.0 ± 4.1, CON 6.3 ± 4.9 [μmol]; *p* = 0.011, η_p_^2^ = 0.274; StO_2_: COMP 3.5 ± 1.9, PLAC 2.4 ± 1.2, CON 2.3 ± 1.9 [%]; *p* = 0.028, η_p_^2^ = 0.225) phases of hand grip measurements, whereas total [heme] concentrations were not affected. No differences were detected between the conditions for the parameters of peak force and fatigue index in the hand grip, time to failure and hemodynamics in the finger hang, or performance-related parameters in the lap climbing measurements (*p* ≤ 0.05).

**Conclusions:** Forearm compression sleeves did not enhance hand grip strength and endurance, sports climbing performance parameters, physiological responses, or perceptual measures. However, they did result in slightly more pronounced changes of oxy [heme] and StO_2_ in the deoxygenation and reoxygenation phases during the hand grip strength and endurance measurements.

## Introduction

Wearing compression garments is a popular intervention used by recreational and elite athletes to improve current or subsequent exercise performance, reduce the risk of injury, and mitigate exercise-induced discomfort (MacRae et al., 2011; Beliard et al., 2015). Compression garments are elastic clothing items that apply mechanical pressure at the surface of needed body zones, thereby improving venous return and stabilizing, compressing, and supporting the underlying tissues (Bochmann et al., 2005; MacRae et al., 2011; Xiong and Tao, 2018). The use of lower body and lower limb compression garments as a recovery tool has gained popularity both during and after exercise, and the beneficial effects of compression garments on recovery mechanisms are well investigated (MacRae et al., 2011; Hill et al., 2014; Marqués-Jiménez et al., 2016; Brown et al., 2017).

However, evidence remains controversial regarding the beneficial effects on muscle strength and muscle endurance when compression garments are applied during exercise (Born et al., 2013; Beliard et al., 2015; Engel and Sperlich, 2016; Ballmann et al., 2019). The advantages of wearing compression garments during exercise for an enhancement of exercise performance have largely been studied in endurance-based aerobic activities, and the available studies have produced mixed results (Dascombe et al., 2011; Born et al., 2013; Driller and Halson, 2013; Engel et al., 2016). Consequently, evidence for an improvement in exercise performance by wearing compression garments in high-intensity exercise is still insufficient (Ballmann et al., 2019). A few studies have proposed the effectiveness of compression garment application in improving muscle blood flow and repeated sprint cycling performance (Broatch et al., 2018), high-intensity intermittent exercise performance (Sear et al., 2010), or high-intensity exercise performance in basketball players (Ballmann et al., 2019). Wearing compression garments also reduced fatigue-induced strength loss after 100 maximal isokinetic eccentric contractions (Négyesi et al., 2021) and reduced the maximal voluntary contraction force decrements observed immediately after handball-specific exercise circuits that included intermittent sprints, jumps, and agility drills (Ravier et al., 2018). Another investigation reported that wearing compression socks during high-intensity running had a positive impact on subsequent running performance (Brophy-Williams et al., 2019). Conversely, although compression garments lead to a lower effort perception and a reduced self-reported muscle soreness, they are not found to result in performance changes in team-sport activities (Duffield et al., 2008), the 400 m sprint (Faulkner et al., 2013), or during high intensity exercise (Da Silva et al., 2018). Given these contradictory results, further studies are needed to investigate the effects of compression garments on high-intensity exercise performance with relation to specific sports disciplines.

Climbing performance is highly dependent on the high-intensity exercise performance of the finger flexor muscles (Watts et al., 1993; España-Romero et al., 2009; Vigouroux et al., 2015). In general, climbing consists of an acyclic movement pattern that requires repeated isometric contractions of the forearms, combined with dynamic whole-body movements (Baláš et al., 2016). Thus, intermittent finger flexor muscle strength and muscle endurance are key elements of sport-climbing performance (Michailov, 2014; Fryer et al., 2015), and climbing performance and failure in competitive climbing tasks are often related to muscle fatigue of the finger flexor muscles (Watts et al., 1993; Philippe et al., 2012).

During climbing, handgrip strength and endurance decrease significantly because of muscle contraction-induced ischemia in the finger flexor muscles. This ischemia is associated with a decline in muscle oxygenation and results in muscle fatigue and performance decrements (Watts, 2004; Fryer et al., 2016; Engel et al., 2018). The forearm flexor oxidative capacity index is another important determinant of rock climbing performance (Fryer et al., 2016). External forearm compression, induced by wearing forearm compression sleeves, has been shown to increase forearm arterial blood flow (Bochmann et al., 2005); therefore, this enhanced blood flow could be beneficial for sports climbing performance. The enhanced flow is associated with an increased ability of all tissues (including working muscles) to utilize lactate, the metabolic response to an increased glycolytic flux elicited by an increasing work rate (Giles et al., 2006; Poole et al., 2021).

Although the beneficial effects of compression garments remain uncertain for high-intensity performance and the improvement of muscular strength and endurance, a broad interest exists, from a practical point of view, in the potential benefits of wearing compression garments in complex sport tasks, such as sports climbing. At present, only one study has investigated the effects of wearing forearm compression sleeves on climbing performance (Engel et al., 2018). This recent study suggested the occurrence of a compression-induced improvement in oxygenation and reduction in ischemia, but no changes were evident in blood lactate concentrations, heart rates, rates of perceived exertion, or local muscle pain in the forearms when elite bouldering athletes wore forearm compression sleeves during and after a set of severe climbing (Engel et al., 2018). Therefore, further studies are needed to confirm the potential effects of compression garments on sports climbing performance. The aim of our study was to examine the immediate effects of the use of forearm compression sleeves on sports climbing performance in recreationally trained sports climbers. We hypothesized that wearing forearm compression sleeves over the finger flexor muscles would affect muscle strength and endurance and would be reflected in altered sports climbing performance-related parameters.

## Materials and Methods

### Experimental Design

**FIGURE 1:**
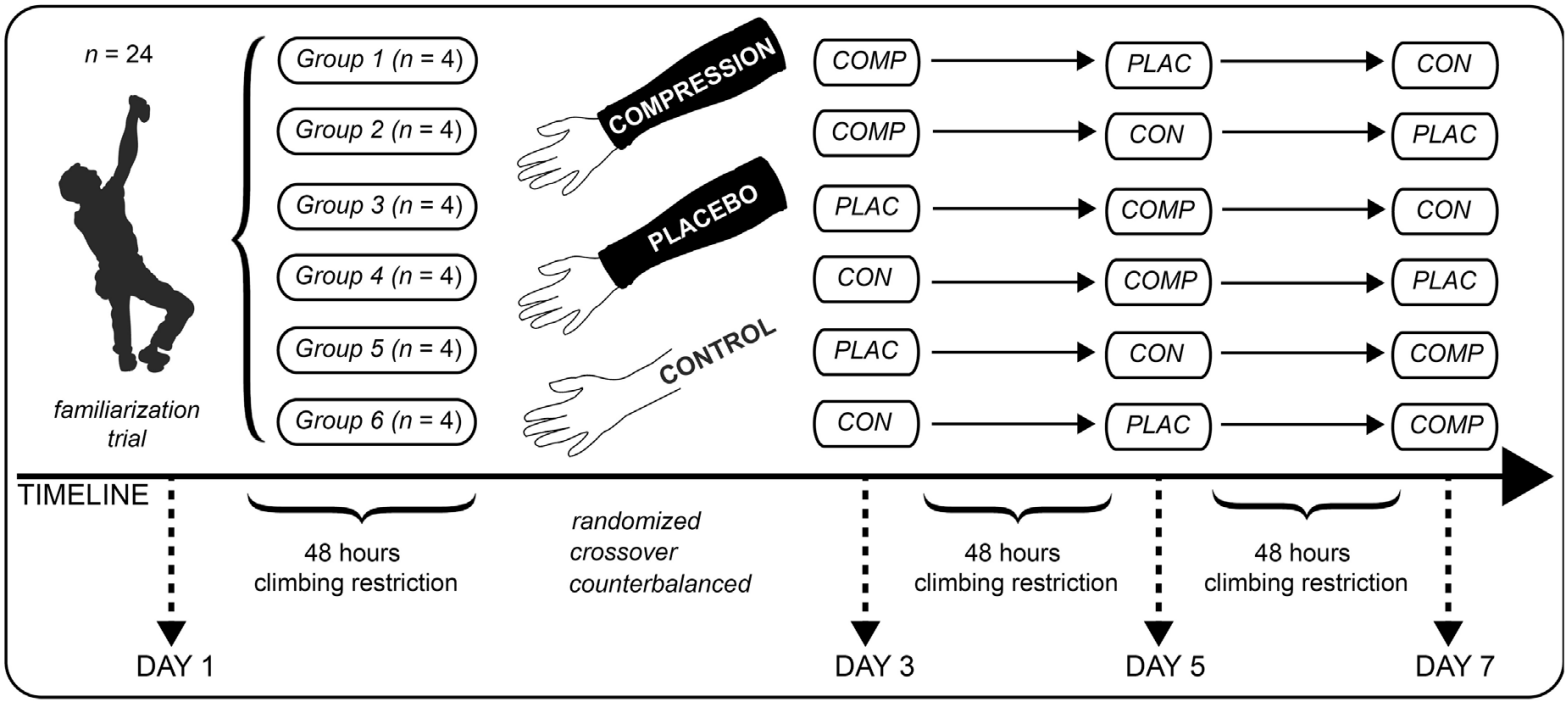
Experimental design.

The present study was conducted as a double-blind, counterbalanced, placebo-controlled crossover study. The experimental protocol was completed in 4 visits, separated by at least 48 h and not more than 72 h (Figure 1). All test trials were conducted on an indoor climbing wall and the evaluation of each participant took place at approximately the same time of day to minimize the effects of diurnal variations on the measured variables. The interference of uncontrolled variables was reduced by instructing all participants not to exercise within 48 h before the test trials and to avoid alcohol and caffeine ingestion during the experimental period. Participants were asked to maintain their normal dietary habits and habitual lifestyle before and during the experimental period.

Before beginning each assessment, each participant completed a standardized 10 min warm-up that included a general activation of the cardiovascular system, coordination, and light dynamic stretching. The first visit served as a familiarization trial for the test procedures and for determination of the onsight climbing ability, and each participant was advised of the purpose, benefits, and risks associated with the investigation. During the familiarization trial, the body weight and body fat percentage were determined with a digital scale (Tanita BC 601; Tanita Europe, Amsterdam, Netherlands) and height was measured (to the nearest 0.1 cm) using a stadiometer. The onsight climbing ability was determined by first asking the subjects about their most difficult ascent by top rope or red point style, whichever was higher, rated on the UIAA (Union Internationale des Associations d’Alpinisme) scale. The participants then had to climb a predefined route at the self-reported level of difficulty in top rope style. If this route was accomplished without falling, the route difficulty was increased for the next route until no better attempt was possible. If the predefined route was not accomplished, the degree was decreased accordingly for the next attempt. A break of 20 min was given between the attempts. The best attempt was rated as the participant’s onsight climbing performance within this study. The UIAA scale for free climbing difficulty currently extends from I to XII (Draper et al., 2016). For statistical analyses and a better international comparability, the UIAA climbing scale was converted to the metric IRCRA (International Rock Climbing Research Association) scale, according to recommendations for the statistical analysis of sports-climbing grades (Watts et al., 1993; Draper et al., 2016). On the following three visits, the participants completed the test trials in a crossover design, either with compression forearm sleeves (COMP), with non-compressive placebo forearm sleeves (PLAC), or without forearm sleeves (CON). The three test trials (COMP, PLAC, and CON) included three different measurements (hand grip endurance, finger hang, and lap climbing) after completion of the standardized 10 min warm-up. Between the measurements, each subject was required to take a 10 min rest break.

### Participants

An *a priori* power calculation indicated that 21 participants were needed to detect significant differences between the conditions COMP, PLAC, and CON, based on an estimated α level of 0.05 and a power of 90% (based on blood lactate concentration results after maximum-intensity climbing bouts (forearm compression sleeves vs placebo sleeves) from an earlier study with an effect size of η_p_^2^ = 0.10 (Engel et al., 2018)). A total of 26 (13 male, 13 female) recreationally and moderately trained climbers volunteered to participate in this study. Of those, two dropped out during the study due to busy schedules. The results presented were obtained from the remaining 24 participants (12 male, 12 female). The participants had a mean (±SD) age of 29.1 ± 6.6 years and showed climbing abilities ranging from IRCRA 13 to 18 (14.8 ± 1.4). Characteristics of male and female participants are shown in Table 1.

**TABLE 1:** Anthropometric data and climbing ability of male (*n* = 12) and female (*n* = 12) participants.

	Male (*n* = 12)	Female (*n* = 12)
Age (years)	30.0 ± 7.2	28.2 ± 6.2
Body mass (kg)	74.8 ± 10.2	59.4 ± 5.6
Height (cm)	180.8 ± 6.1	166.6 ± 5.8
BMI (kg/m2)	22.8 ± 2.3	21.4 ± 1.0
Body fat (%)	9.7 ± 3.7	16.5 ± 4.2
CA (IRCRA)	15.3 ± 1.6	14.3 ± 1.0
Forearm circumference (cm)	28.8 ± 1.9	24.6 ± 1.0

Note: Data is presented as mean ± standard deviation of the mean.

BMI, body mass index. Climbing ability (CA) is indicated according to the IRCRA (International Rock Climbing Research Association) scale. For further details see Section 2.

All participants underwent a medical screening before entering the study. Participants had to be in good health, with no pre-existing cardiac or pulmonary conditions. Exclusion criteria included acute infections, alcohol consumption at any time during the test period, and chronic medication intake, as well as acute muscular injuries or restrictions. Inclusion criteria were a top rope onsight climbing ability (CA) of at least UIAA grade VII- (IRCRA 13), at least 3 years of climbing experience, an average training load of at least 2 days per week with 3 h per session within the last 3 months, and a self-report of sports climbing as a predominant discipline. All participants gave their written informed consent prior to participation, and all procedures were approved by the ethical committee of the German Sports University Cologne in accordance with the Declaration of Helsinki.

### Compression Sleeves

During warm-up and the test trials, the participants wore either compression forearm sleeves (VERTICS.Sleeves, Vertics, Wiesbaden, Germany), non-compressive placebo forearm sleeves (Kiprun Forearm Sleeves, Kalenji/Decathlon S.A., Villeneuve d'Ascq, France), or no forearm sleeves (as control condition). The compression forearm sleeves were made from a 75% polyamide and 25% spandex fabric and exerted a graduated compression from distal (22.4 mmHg) to proximal (12.4 mmHg) (Engel et al., 2018). The placebo forearm sleeves consisted of 83% polyester, 13% polyamide, and 4% spandex and were non-compressive.

The placebo and graduated compression sleeves were visually similar, and the participants were informed that the effectiveness of the two types of forearm sleeves was being tested; they remained unaware of differences in capacity for compression. The investigators were informed about the capacity of compression of both forearm sleeves. The forearm circumference was used to select the size of the forearm sleeves in accordance with the manufacturer’s instructions.

### Hand Grip Strength and Endurance

An adjustable Jamar^®^ Plus+ (Patterson Medical/Sammons Preston, Illinois, United States) digital hand dynamometer was used for handgrip measurements. The participants were seated, without leaning, with an elbow flexion at 90°, a slight shoulder abduction of about 15°, and with their forearms in a neutral supination/pronation position (Trampisch et al., 2012). For the hand grip endurance measurement, an audible signal was given every 3 s to indicate the change between maximal effort and relaxation. Each participant was required to complete 10 trials of maximum voluntary contraction (MVC) (Ozimek et al., 2016). MVC had to be built up from zero to maximum effort within 3 s and was followed by a 3 s rest for each trial. The participants were asked to grip the handle of the dynamometer with maximum effort and were verbally encouraged throughout the test. We determined the following variables over the 10 MVC trials: peak force (F_max_), lowest force (F_min_), and fatigue index (FI). The F_max_ and F_min_ were defined as the highest and lowest forces, respectively, achieved within the 10 MVC trials, whereas FI was calculated as FI (%) = [(F_max_–F_min_)/F_max_] × 100. The variables F_max_ and FI were used for further statistical analyses. We eliminated a potential hand dominance by averaging the right and left hand values for F_max_ and FI, and both variables were defined as non-specific muscle endurance parameter dominance (Watts, 2003; Baláš et al., 2012; Kim and Kim, 2016).

### Finger Hang

The participants’ muscle endurance (resistance to fatigue) of the finger flexors was assessed by having the climbers hold onto a 4 cm ledge with their arms straight (Ozimek et al., 2016). The hold on the ledge was carried out with four fingers in an open grip; the thumb was not included in the grip and was positioned at the bottom of the ledge (Baláš et al., 2012). The elbow, shoulder, and hip joints had to remain fully extended while the participant was hanging on the ledge. The time to failure was determined as the point when the climber could no longer maintain this position on the ledge and was defined as a specific muscle endurance parameter (hang time in seconds).

### Lap Climbing

The third measurement was lap climbing (LC) to assess each participant’s sports climbing endurance performance. In the LC test, the participants were required to climb a predefined route as many times as possible on top rope belay, with no rest between the ascents. The route was set at one UIAA grade below the assessed maximum onsight climbing level (climbing ability VIII > LC route VII). The climbing routes were chosen on a 15 m high vertical to slightly overhanging wall. Only homogeneous, mainly regular, and topographically similar routes were included to induce high levels of perceived exertion and muscle fatigue (Button et al., 2018). The climbing routes consisted of climbing hand holds, with only a few differences in the types of hand holds. The route difficulty was established mainly with buckets, ledges, and crimps. Only routes without pronounced “cruxes” (demanding regions of the route where more advanced climbing actions could be required for further progress) were included in the LC test (Button et al., 2018). The potential influence of recovery strategies on climbing performance was standardized by forbidding the participants from chalking or shaking their hands during climbing or lowering (Baláš et al., 2016). The LC was to be accomplished until a fall occurred due to fatigue. The total covered climbing distance (LCD) in m and the climbing time (LCT) in min were assessed by video analysis. Once the subject had to cease due to fatigue, capillary blood samples (20 μL) were collected from the earlobe directly and after 2, 4, 6, and 8 min after the LC trials to assess blood lactate values. Capillary blood samples were taken while the participants were sitting, without any further physical activity, next to the climbing wall. Blood lactate measurements were conducted directly after the LC trials (Biosen S-Line, EKF-diagnostic GmbH, Magdeburg, Germany). The maximum post-exercise lactate concentration (La_max_) was used for statistical analyses. The rate of perceived exertion (RPE) and forearm muscle pain were also assessed using Borgs’ RPE scale (Borg, 1982) and the visual analog (VAS) graphic rating scale (Lee et al., 1991), respectively.

### Near-Infrared Spectroscopy

Near-infrared spectroscopy was assessed during the hand grip strength and endurance measurements, as well as during the finger hang. The oxygenation and blood volume changes were assessed using a continuous-wave near-infrared (NIR) spectrophotometer (NIRS; OxyMon MKIII, Artinis Medical System, BV, Elst, Netherlands) and Oxysoft software (Artinis Medical System, BV). The OxyMon MK III instrument generates NIR light with wavelengths at 765 and 855 nm; a sampling rate of 10 Hz and a fixed differential path-length factor of 4.0 were used, as suggested by van Beekvelt et al. (2002). The Lambert-Beer law, in which a path-length factor is incorporated to correct for scattering of light in the tissue, was used to convert the changes in absorption at the discrete wavelengths into concentration changes in oxygenated and deoxygenated hemoglobin/myoglobin/cytochrome oxidase (Barstow, 2019). The spectrum of these hemes compounds overlaps, so differentiation between changes was not possible using NIRS measurements. However, since exercise performance studies are mainly interested in the total amount of O_2_ consumed during exercise, the results are not affected by this aspect (van Beekvelt et al., 2002). We therefore reported the sum of the main absorbing chromophores in skeletal muscle as [heme] in the present study, following the suggestion of Barstow (2019).

The flexor digitorum profundus (FDP) muscle was chosen for NIRS measurements because it has been proposed as the most important forearm muscle for sports climbing performance (Michailov, 2014; Fryer et al., 2015) and has been used in previous research on hemodynamics in sports climbers (Philippe et al., 2012; Fryer et al., 2015; Baláš et al., 2018; Feldmann et al., 2020). NIRS optodes were placed between the medial epicondyle of the humerus and the styloid process of the ulna at 1/3 of the proximal distance (Schweizer and Hudek, 2011; Baláš et al., 2018). Following recent recommendations for the location of the FDP (Fryer et al., 2016), the participants were asked to squeeze their thumbs and the first fingers together, and the investigator then palpated the FDP muscle to locate the middle of the muscle belly. The specific point was marked with a skin-friendly permanent pen to avoid variations in the placement of the optodes over all test trials.

The optodes were placed in a template holder on the skin with an interoptode distance of 40 mm, and the template holder was fixed to the skin using dark opaque tape to prevent possible ambient light interference. The effectiveness of the optodes is affected by the presence of excessive adipose tissue in the body (van Beekvelt et al., 2001). However, all participants had a generally low percentage body fat (Table 1), and the forearms are not a major site of subcutaneous body fat. Therefore, we assumed that subcutaneous adipose tissue thickness did not interfere with data collection (Fryer et al., 2015).

**FIGURE 2:**
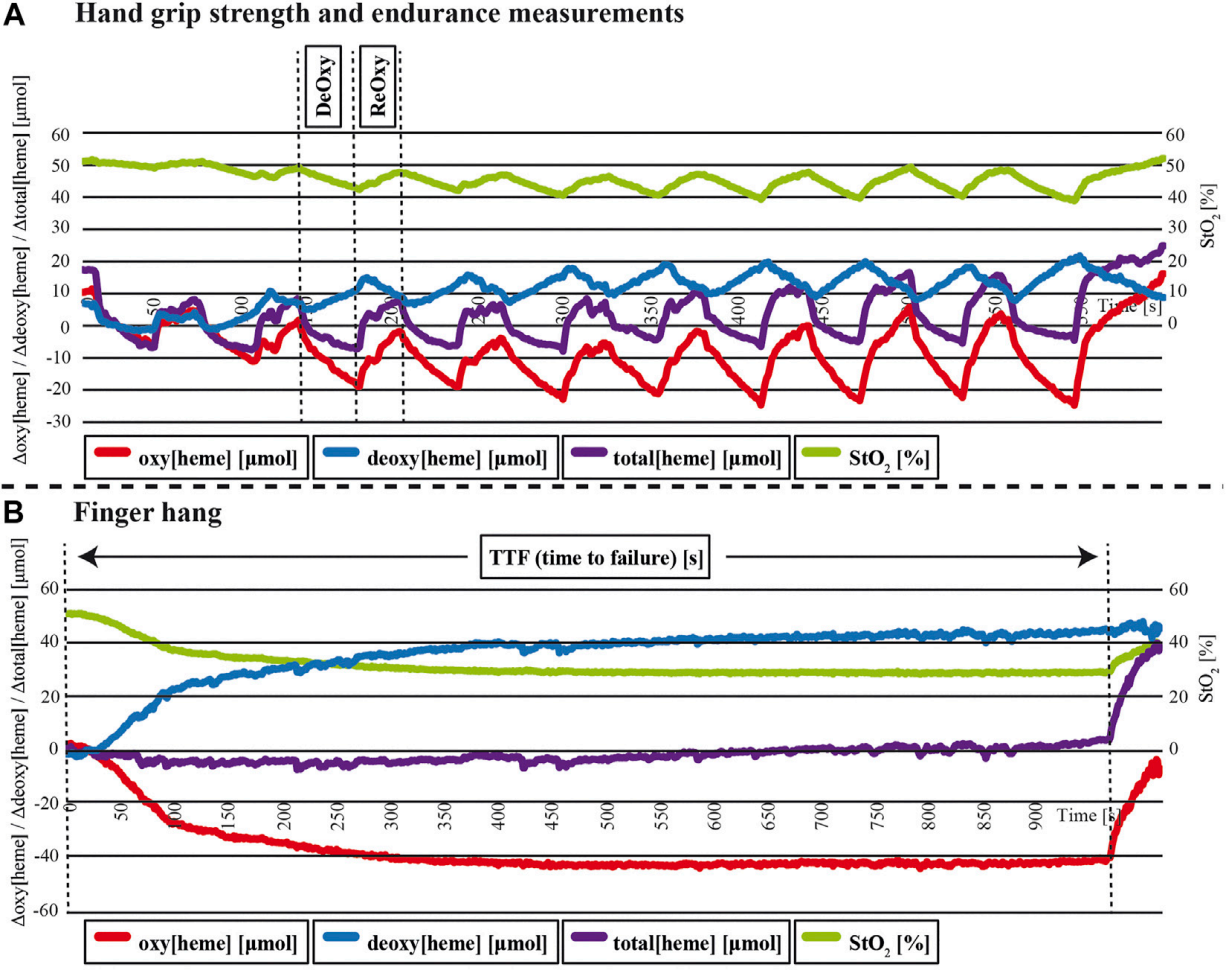
Representative changes in tissue oxygen saturation (StO_2_), oxygenated (oxy[heme]), deoxygenated (deoxy[heme]), and total (total[heme]) [heme] during hand grip strength and endurance **(A)** and finger hang **(B)** measurements.

The NIRS outcome measures were oxygenated [heme] (oxy[heme]) and deoxygenated [heme] (deoxy[heme]) concentrations. The sum of the oxy[heme] and deoxy[heme] values were the total [heme] concentrations (total[heme]), and the tissue oxygen saturation (StO_2_) was calculated as oxy[heme]/(deoxy[heme] + oxy[heme]). In accordance with the approach reported in earlier studies (MacLeod et al., 2007; Philippe et al., 2012; Fryer et al., 2015; Baláš et al., 2018), during the hand grip strength and endurance measurements, the changes in the tissue oxygenation and blood volume (ΔStO_2_, Δoxy [heme], Δtotal[heme]) were calculated from the maximum concentrations of StO_2_, oxy[heme], and total[heme], while the minimum concentrations were used to represent the mean deoxygenation in the 3 s contraction periods and the mean reoxygenation in the 3 s relief periods (Figure 2A). The mean deoxygenation (ΔStO_2Deoxy_, Δoxy[heme]_Deoxy_, Δtotal[heme]_Deoxy_) and reoxygenation (ΔStO_2Reoxy_, Δoxy [heme]_Reoxy_, Δtotal[heme]_Reoxy_) concentration changes were calculated over all 10 MVC trials. Because of missing NIRS data, the results of the variables ΔStO_2Deoxy_, Δoxy[heme]_Deoxy_, Δtotal[heme]_Deoxy_, ΔStO_2Reoxy_, Δoxy[heme]_Reoxy_, and Δtotal[heme]_Reoxy_ in hand grip strength and endurance measurements are presented for the remaining 16 full data sets only.

The statistical analyses of the continuous finger hang incorporated the changes in tissue oxygenation and blood volume during the continuous finger hang exercise (ΔStO_2_, Δoxy[heme], Δtotal[heme]) for all 24 full data sets. The changes were calculated as the decrease from the maximum concentrations of StO_2_, oxy[heme], and total[heme] at the beginning of the finger hang to the minimum concentration (MacLeod et al., 2007) (Figure 2B).

### Statistical Analyses

Data are presented as means ± standard deviations. The Shapiro-Wilk test was used to identify all departures from normal distribution. The effects of treatments (COMP, PLAC, and CON) on the parameters F_max_, FI, hang time, LCD, LCT, La_max_, and all NIRS output parameters were tested by one-way repeated measures ANOVA, with sex (male or female) as a between subject factor. Violations of the assumption of sphericity were corrected using Greenhouse–Geisser adjustments. Two-tailed paired *t*-tests were utilized as *post hoc* tests to indicate significant differences. A Bonferroni procedure was used (p*) to retain an α = 0.05, and the significance level was set at *p* ≤ 0.05 for all comparisons. Effect sizes were calculated using partial η squared (η_p_^2^), and were interpreted as small (0.01), medium (0.06), and large (0.14). For the ordinal parameters RPE and VAS, the Friedman test was used to identify differences between treatments (COMP, PLAC, CON). Dunn-Bonferroni tests were used for post-hoc comparisons of the means with corrections for multiple tests to retain an alpha level of 0.05. Kendall’s *W* (coefficient of concordance) was used to interpret the effect sizes. Student’s *t*-test was calculated for differences in IRCRA climbing ability between female and male participants and Cohen’s d (*d*) was used to calculate effect size. The alpha level was set at *p* ≤ 0.05, and all analyses were conducted using SPSS 28 (IBM Corp., Armonk, NY, United States).

## Results

### Hand Grip Strength and Endurance

**FIGURE 3:**
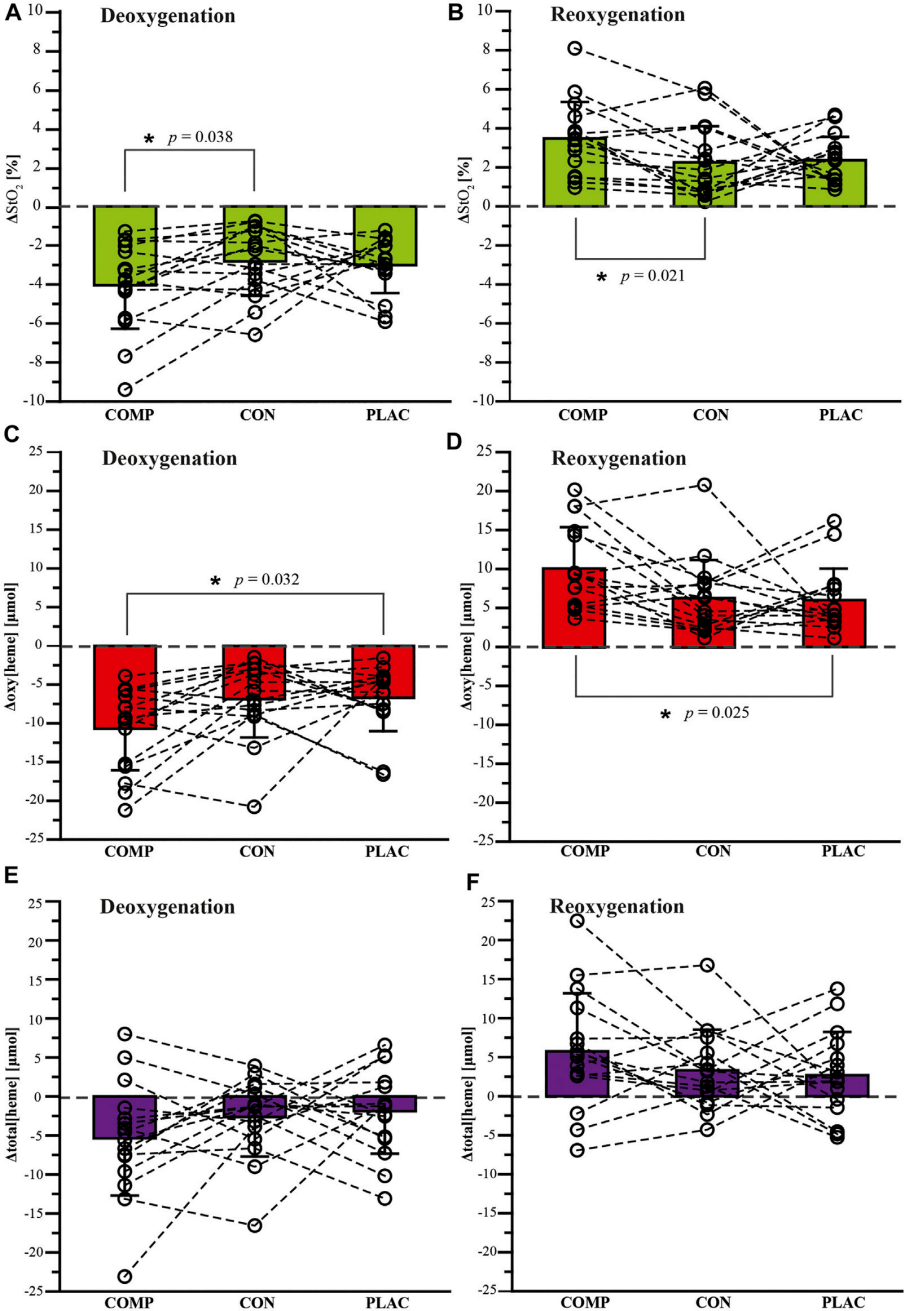
Near-infrared spectroscopy (NIRS) changes during hand grip strength and endurance measurements (*n* = 16) under compression (COMP), control (CON), and placebo (PLAC) conditions for: **(A,B)** tissue oxygen saturation (ΔStO_2_), **(C,D)** oxygenated (Δoxy[heme]), and **(E,F)** total (total [heme]) [heme]. Data points represent individual values (○). Bar charts are means ± SD. See the Section 2 for further details, *p < 0.05.

We found no significant main treatment effects for F_max_ (*p* = 0.442, η_p_^2^ = 0.036) and FI (*p* = 0.695, η_p_^2^ = 0.016) (Table 2). The condition sex also had no influence on the intervention-induced changes in F_max_ (*p* = 0.384, η_p_^2^ = 0.043) and FI (*p* = 0.349, η_p_^2^ = 0.047), but the F_max_ values were significantly lower for female than for male participants (*p* < 0.001, η_p_^2^ = 0.542) (Table 2). Conversely, the IRCRA climbing abilities did not differ between female and male participants (female 14.3 ± 1.0, male 15.3 ± 1.6; *p* = 0.070, *d* = –0.776). We also found a significant main treatment effect for the NIRS-related parameters ΔStO_2Deoxy_ (COMP –4.0 ± 2.2, PLAC –3.0 ± 1.4, CON –2.8 ± 1.8 [%]; *p* = 0.049, η_p_^2^ = 0.194), Δoxy[heme]_Deoxy_ (COMP –10.7 ± 5.4, PLAC –6.7 ± 4.3, CON –6.9 ± 5.0 [μmol]; *p* = 0.014, η_p_^2^ = 0.263), ΔStO_2Reoxy_ (COMP 3.5 ± 1.9, PLAC 2.4 ± 1.2, CON 2.3 ± 1.9 [%]; *p* = 0.028, η_p_^2^ = 0.225), and Δoxy[heme]_Reoxy_ (COMP 10.2 ± 5.3, PLAC 6.0 ± 4.1, CON 6.3 ± 4.9 [μmol]; *p* = 0.011, η_p_^2^ = 0.274) (Figures 3A–D). The parameters Δtotal [heme]_Deoxy_ (COMP –5.4 ± 7.4, PLAC –1.9 ± 5.4, CON –2.6 ± 5.1 [μmol]; *p* = 0.302, η_p_^2^ = 0.082) and Δtotal[heme]_Reoxy_ (COMP 5.8 ± 7.4, PLAC 2.7 ± 5.6, CON 3.3 ± 5.2 [μmol]; *p* = 0.424, η_p_^2^ = 0.059) had no significant differences (Figures 3E,F). The condition sex showed no influence on NIRS-related parameters in hand grip strength and endurance measurements (ΔStO_2Deoxy_: *p* = 0.123, η_p_^2^ = 0.139; Δoxy[heme]_Deoxy_: *p* = 0.352, η_p_^2^ = 0.072; Δtotal[heme]_Deoxy_: *p* = 0.774, η_p_^2^ = 0.018; ΔStO_2Reoxy_: *p* = 0.090, η_p_^2^ = 0.158; Δoxy[heme]_Reoxy_: *p* = 0.364, η_p_^2^ = 0.070; Δtotal[heme]_Reoxy_: *p* = 0.573, η_p_^2^ = 0.039) (for data and pairwise comparisons see Table 3).

**TABLE 2:** Performance outputs for intermittent hand grip strength and endurance measurements of female (*n* = 12), male (*n* = 12), and all (*n* = 24) participants.

		COMP	CON	PLAC	*p*	η_p_^2^
*F*_*max*_ *(N)*	female	332.7 ± 51.5*	344.2 ± 56.8*	334.5 ± 48.1*	*<0.001*	0.542
	male	485.8 ± 95.0	488.9 ± 97.0	496.0 ± 87.0		
	all	409.3 ± 108.2	416.5 ± 107.2	415.3 ± 107.4	0.442	0.036
*FI (%)*	female	32.1 ± 7.0	30.1 ± 9.2	29.0 ± 9.6	0.764	0.004
	male	29.3 ± 5.1	29.3 ± 4.7	30.3 ± 6.5		
	all	30.7 ± 6.2	29.7 ± 7.1	29.6 ± 8.1	0.695	0.016

Note: Data are presented as mean ± standard deviation of the mean.

COMP, compression trial; CON, control trial; PLAC, placebo trial; F_max_, peak force; FI, fatigue index; asterisks (*) indicate significant differences compared to male participants.

*p* and η_p_^2^ indicate pairwise comparisons between female and male participants (“*male*”, “*female*”), and main treatment effects for the conditions COMP, CON, and PLAC (“*all*”). For further details see the Section 2.

**TABLE 3:** Near-infrared spectroscopy changes and pairwise comparisons during intermittent hand grip strength and endurance measurements of female (*n* = 10) and male (*n* = 6) participants.

			COMP	CON	PLAC	*p*	η_p_^2^
ΔStO_2_ (%)	DeOxy	female	–3.4 ± 2.1	–2.4 ± 1.9	–3.2 ± 1.7	0.243	0.096
		male	–5.1 ± 2.3	–3.6 ± 1.4	–2.6 ± 0.7		
	ReOxy	female	2.8 ± 1.6	1.8 ± 1.7	2.6 ± 1.4	0.200	0.114
		male	4.5 ± 1.9	3.0 ± 2.0	1.9 ± 0.7		
Δoxy[heme] (μmol)	DeOxy	female	–10.0 ± 5.5	–6.2 ± 5.9	–7.5 ± 5.3	0.777	0.006
		male	–11.9 ± 5.5	–8.0 ± 3.0	–5.4 ± 1.3		
	ReOxy	female	9.5 ± 5.5	5.8 ± 5.9	6.8 ± 5.1	0.863	0.002
		male	11.2 ± 5.4	7.3 ± 2.9	4.6 ± 1.1		
Δtotal[heme] (μmol)	DeOxy	female	–6.6 ± 3.8	–3.1 ± 5.3	–2.1 ± 6.2	0.432	0.045
		male	–3.3 ± 11.3	–1.9 ± 5.1	–1.6 ± 4.5		
	ReOxy	female	7.2 ± 4.7	3.7 ± 5.3	2.6 ± 6.3	0.493	0.034
		male	3.3 ± 10.7	2.7 ± 5.5	2.8 ± 4.7		

Note: Data are presented as mean ± standard deviation of the mean.

COMP, compression trial; CON, control trial; PLAC, placebo trial; StO_2_, tissue oxygen saturation; oxy[heme], oxygenated [heme]; Δ, mean decrease (DeOxy = deoxygenation) and increase (ReOxy = reoxygenation) from the contraction and release phases of hand grip measurement; *p* and η_p_^2^ indicate pairwise comparisons between female and male participants; For further details see the Section 2.

### Finger Hang

No significant main treatment effect was detected for the time to failure in the finger hang (*p* = 0.327, η_p_^2^ = 0.049) and the condition sex had no influence on the changes in hang time (*p* = 0.955, η_p_^2^ = 0.002) (Table 4). We also found no significant main treatment effect for the NIRS-related parameters ΔStO_2_, Δoxy[heme], and Δtotal[heme] (Table 4). The condition sex showed no influence on NIRS-related parameters in the finger hang for ΔStO_2_ (*p* = 0.790, η_p_^2^ = 0.011), Δoxy[heme] (*p* = 0.887, η_p_^2^ = 0.005), and Δtotal[heme] (*p* = 0.589, η_p_^2^ = 0.024) (Table 4).

**TABLE 4:** Time to failure and near-infrared spectroscopy changes during continuous finger hang tests of female (*n* = 12), male (*n* = 12), and all (*n* = 24) participants.

		COMP	CON	PLAC	*p*	η_p_^2^
TTF (s)	female	74.8 ± 23.8	73.6 ± 23.5	77.8 ± 19.7	0.882	0.001
	male	75.3 ± 19.6	75.7 ± 16.6	79.0 ± 28.7		
	all	75.0 ± 21.4	74.7 ± 19.9	78.4 ± 24.1	0.327	0.049
ΔStO_2_ (%)	female	–19.9 ± 11.0	–16.6 ± 7.9	–18.4 ± 11.7	0.242	0.062
	male	–24.2 ± 10.7	–22.2 ± 11.7	–20.6 ± 11.2		
	all	–22.0 ± 10.8	–19.4 ± 10.2	–19.5 ± 11.3	0.488	0.032
Δoxy[heme] (μmol)	female	–20.2 ± 12.0	–19.4 ± 11.1	–17.6 ± 11.5	0.313	0.046
	male	–25.2 ± 10.8	–22.4 ± 13.6	–21.7 ± 13.0		
	all	–22.7 ± 11.5	–20.9 ± 12.2	–19.7 ± 12.2	0.512	0.030
Δtotal[heme] (μmol)	female	–1.9 ± 7.1	–1.7 ± 6.2	2.0 ± 4.8	0.592	0.013
	male	–1.6 ± 8.0	–1.7 ± 12.1	–2.0 ± 11.3		
	all	–1.7 ± 7.4	–1.7 ± 9.4	–0.0 ± 8.7	0.709	0.016

Note: Data are presented as mean ± standard deviation of the mean.

COMP, compression trial; CON, control trial; PLAC, placebo trial; TTF, time to failure, StO_2_, tissue oxygen saturation; oxy[heme], oxygenated [heme]; Δ, decrease from the maximum concentrations at the beginning of the finger hang to the concentration at the time point of task failure; *p* and η_p_^2^ indicate pairwise comparisons between female and male participants (“*male*”, “*female*”), and main treatment effects for the conditions COMP, CON, and PLAC (“*all*”). For further details see the Section 2.

### Lap Climbing

We found no significant main treatment effects for LCT (*p* = 0.169, η_p_^2^ = 0.078), LCD (*p* = 0.188, η_p_^2^ = 0.073), and La_max_ (*p* = 0.245, η_p_^2^ = 0.062) (Table 5). The condition sex had no influence on changes in LCT (*p* = 0.210, η_p_^2^ = 0.068), or LCD (*p* = 0.147, η_p_^2^ = 0.084), or La_max_ (*p* = 0.858, η_p_^2^ = 0.007). A further comparison of treatments revealed no influence of the wearing of compression forearm sleeves on RPE (*p* = 0.950, *W* = 0.002) or VAS (*p* = 0.431, *W* = 0.035) (Table 5).

**TABLE 5:** Lap climbing performance measurements of female (*n* = 12), male (*n* = 12), and all (*n* = 24) participants.

		COMP	CON	PLAC	*p*	η_p_^2^/*W*
LCT (s)	female	484 ± 197	495 ± 218	575 ± 414	0.847	0.002
	male	421 ± 133	584 ± 321	496 ± 203		
	all	452 ± 167	540 ± 272	535 ± 321	0.169	0.078
LCD (m)	female	74.9 ± 42.7	72.7 ± 47.8	88.2 ± 82.2	0.502	0.021
	male	74.6 ± 25.5	106.8 ± 55.5	91.9 ± 41.5		
	all	74.7 ± 34.4	89.7 ± 53.5	90.0 ± 63.7	0.188	0.073
La_max_ (mmol/l)	female	7.05 ± 2.29	7.77 ± 2.68	7.01 ± 2.15	0.391	0.034
	male	7.71 ± 2.68	8.34 ± 2.55	8.01 ± 2.81		
	all	7.38 ± 2.02	8.05 ± 2.57	7.51 ± 2.50	0.245	0.062
RPE	female	15.9 ± 2.3	16.2 ± 2.1	16.3 ± 2.0	0.395	0.033
	male	16.8 ± 2.0	16.8 ± 1.4	16.6 ± 1.5		
	all	16.4 ± 2.1	16.5 ± 1.8	16.5 ± 1.7	0.950	0.002
VAS	female	6.1 ± 1.7	6.3 ± 1.9	5.8 ± 1.9	0.964	0.000
	male	6.3 ± 1.7	6.0 ± 1.6	5.8 ± 1.6		
	all	6.2 ± 1.7	6.2 ± 1.7	5.8 ± 1.7	0.431	0.035

Note: Data is presented as mean ± standard deviation of the mean.

COMP, compression trial; CON, control trial; PLAC, placebo trial; LCT, lap climbing time; LCD, lap climbing distance; La_max_, maximum blood lactate concentration; RPE, Borgs’ rate of perceived exertion; VAS, visual analog scale; *p* and η_p_^2^ indicate pairwise comparisons between female and male participants (“*male*”, “*female*”), and main treatment effects for the conditions COMP, CON, and PLAC (“*all*”). For further details see the Section 2.

## Discussion

The aim of this investigation was to determine whether wearing forearm compression sleeves during exercise has immediate effects on sports climbing performance and diminishes the effects of muscle fatigue. We found no effect of using forearm compression sleeves over the finger flexor muscles on sports climbing-related muscle strength, muscle endurance parameters, maximum blood lactate, or parameters of perceived exertion and muscle pain, but we did note effects on hemodynamic responses. The wearing of forearm compression sleeves resulted in greater changes in oxy[heme] and StO_2_ during the deoxygenation and reoxygenation phases of hand grip strength and endurance measurements, whereas the total [heme] concentrations and hemodynamic changes in finger hang measurements were unaffected. The findings therefore did not clearly support the positive effects on muscle strength and endurance claimed by the manufacturers of forearm compression sleeves and believed in by elite and recreational athletes. The results do, however, suggest that forearm compression sleeves can improve muscle blood flow and tissue saturation.

Our results reflect recent findings of inconsistent beneficial effects when wearing compression sleeves during exercise. Several studies have reported positive effects of sports compression garments on hemodynamics in participants in passive resting positions (Bringard et al., 2006; Lee et al., 2017; O'Riordan et al., 2021) or during exercise (Ménétrier et al., 2011; Broatch et al., 2018). However, compression sleeves often give rise to only a slight increase in tissue oxygen saturation at rest and during recovery from aerobic running exercise, with no influence on time to exhaustion (Ménétrier et al., 2011).

Coza et al. (2012) found changes in tissue blood flow and perfusion in participants wearing calf compression sleeves during short-term dynamic exercise and concluded that these compression-induced changes were a result of improved oxygenation during short-term exercise. They further assumed that the increased muscle oxygen availability positively influenced performance and concluded that compression of muscles may enhance performance, especially in sports that require repeated short bouts of exercise (Coza et al., 2012). Indeed, recent studies suggest positive effects of compression garments on repeated-sprint exercise (Broatch et al., 2018), prolonged high-intensity intermittent exercise (Sear et al., 2010), repeated maximal isokinetic eccentric contractions (Négyesi et al., 2021), and continuous high-intensity performance assessed using the Wingate Anaerobic Test (Ballmann et al., 2019). Conversely, compression garments were found to increase femoral artery diameter, arterial blood flow, and markers of blood oxygen extraction in muscle during repeated-sprint exercise, but they showed no effect on blood lactate or glucose levels on exercise performance (Broatch et al., 2021).

Climbing performance is highly dependent on high-intensity exercise performance and intermittent finger flexor muscle strength and endurance (Michailov, 2014; Fryer et al., 2015; Vigouroux et al., 2015), but neither the study by Engel et al. (2018) nor our present study found beneficial effects of forearm compression sleeves on climbing performance. Even a recent meta-analyses has concluded that wearing lower limb compression garments has only negligible or no effects on performance and physiological responses following high-intensity exercise (Da Silva et al., 2018). However, the results in the present study for hemodynamic changes during repeated hand grip strength measurements support the finding of hemodynamic changes caused by compression garments during high-intensity exercise suggested by Coza et al. (2012).

The lack of evidence for performance-enhancing effects of forearm compression sleeves in sports climbing could arise for several reasons. One is that hemodynamic changes during contraction and relief periods in climbing-specific intermittent tasks are recommended to exceed ∼4.5% for StO_2_ and ∼18.5 mmol for total[heme] to be considered a meaningful change (Baláš et al., 2018). In the present study, we found only small mean changes of ∼3% and ∼4 mmol for StO_2_ and total[heme], respectively, during the intermittent hand grip strength and endurance measurements. The hemodynamic changes may therefore have been insufficient to influence sports climbing-related performance parameters. Although, as presented in Figure 3, large interindividual differences were evident in the hemodynamic responses to wearing forearm compression sleeves, a subgroup might exist that would benefit from the potential effects of compression sleeves. However, our data do not allow us to draw any conclusion regarding the parameters that indicate a higher physiological response. Further research is needed to prove this assumption.

The possibility that wearing forearm compression sleeves may not result in ergogenic effects has also been suggested, as the sleeves cover only a small area and therefore a small percentage of the body surface (Engel et al., 2018). Nevertheless, circulatory and neuromuscular demands in sports climbing rely highly on finger flexor muscles; consequently, ischemia-induced muscle fatigue in the forearm muscles leads to performance decrements in climbing (Schweizer, 2012; Engel et al., 2018). We therefore assume that forearm compression sleeves should be one of the most effective compression garments in sports climbing. However, investigating the effects of full upper body compression garments on sports climbing performance might be interesting in this context, as these other garments cover a higher percentage of the body surface and still include the forearms.

Studies in the existing literature on participants’ sports climbing performance have rarely described the effect of arm compression sleeves on muscle hemodynamics and exercise performance has indeed rarely been described in literature so far. Bochmann et al. (2005) described an increase in forearm arterial blood flow compression induced by wearing forearm compression sleeves at rest and during a simultaneous low-intensity hand grip, but they did not assess actual exercise performance changes. In addition, Pereira et al. (2014) concluded that the use of a graduated arm compression sleeve does not enhance isokinetic elbow flexion muscle performance, but they did not measure oxygenation or blood volume changes. By contrast, wearing a long-sleeved full upper body compression garment resulted in a more maintained external shoulder rotation at 40–50% of maximum voluntary isometric contraction (Tsuruike and Ellenbecker, 2013) and in an enhanced perceptual recovery from manual-labor exercise (Chan et al., 2016). In summary, the research on the wearing of upper limb compression garments and its effects on muscle hemodynamics and exercise performance is still insufficient, especially compared to similar research on lower limb compression aids. Therefore, this might be an interesting area for future investigations.

A second consideration is that the applied graduated compression from distal (22.4 mmHg) to proximal (12.4 mmHg) within our present study might also explain the lack of evidence for performance-enhancing effects of forearm compression sleeves in sports climbing. Differences in pressure levels applied via compression garments are supposed to influence their effectiveness for exercise performance output and to result in different physiological responses (Mizuno et al., 2017; Williams et al., 2020), but this assumption is still questioned (Beliard et al., 2015). The new types of restrictive compression garments have also been viewed as more effective than the more popular graduated compression garments, as they integrate novel resistance technology into compression garments that are now designed to provide variable resistance to movement (Baum et al., 2020).

A further concern is that sports climbing is a highly complex sports task. Successful sports climbing performance depends on maintenance of forearm muscle strength and endurance (España-Romero et al., 2009; Vigouroux et al., 2015; Rokowski et al., 2021), as well as on the physiological components of shoulder and upper body strength and power, core-body and aerobic endurance, flexibility, and balance (MacKenzie et al., 2020; Draper et al., 2021). Psychological and skill-related components, such as route preview, a good climbing movement repertoire, climbing technique, risk management, route management, and mental balance are also required for successful sport-climbing performance (Sanchez et al., 2019; Draper et al., 2021). These additional factors might explain the lack of effects in the present study when recreational climber participants wore forearm compression sleeves while performing sport-climbing activities, so they should be considered in future studies.

A final potential reason for the lack of significant results, and therefore a limitation of the present study, could be that sports compression garments are supposedly more effective during periods of recovery than during actual exercise (Hill et al., 2014; Brown et al., 2017; Cullen et al., 2021). Sports climbing has been an Olympic discipline since 2020; therefore, the enhancement of athletic performance and recovery in climbing has become increasingly important (Engel et al., 2018). The new Olympic combined competition formats, in particular, require high demands from elite athletes. In the Tokyo Olympics in 2021, the single disciplines of lead climbing, bouldering, and speed climbing were combined, and the Paris Olympics in 2024 will include a combined competition of bouldering and lead events, but the speed discipline will be separated. Both combined formats are characterized by long durations, high competition loads, and short recovery periods. Recovery strategies in sports climbing have therefore become increasingly important, and future investigations may focus on compression garments as a recovery aid in sports climbing.

A further limitation of the present study is the choice of test procedures. We decided to combine the less-specific hand grip strength and endurance measurements with the more climbing-specific finger hang and lap climbing measurements. Maciejczyk et al. (2021) proposed that maximal grip force and all-out isometric contractions are equally decisive physical performance indices of climbing performance. However, the continuous finger hang applied in the present study, in particular, does not adequately reflect the requirements of repeated isometric contractions of the forearms in sports climbing (Baláš et al., 2016). Intermittent finger hang tests with alternating contraction and relaxation intervals of 8–10 and 2–3 s are viewed as more climbing-specific and should be implemented in future studies that investigate potential ergogenic compression garment effects in sports climbing (Philippe et al., 2012; Giles et al., 2019; Baláš et al., 2021; Maciejczyk et al., 2021).

We also found significant differences in NIRS outputs between the treatment conditions, but only for the non-specific hand grip strength and endurance measurements, and not for the more climbing-specific finger hang test. We chose the FDP muscle for NIRS measurements because of its importance in climbing-specific performance (Fryer et al., 2016). However, in contrast to climbing performance, hand grip strength and endurance are not mainly dependent on the FDP muscle, but also involve the flexor pollicis longus muscle (Ambike et al., 2014). The lack of effects on NIRS measurements during the finger hang test is therefore unexpected and may be explained by highly different interindividual responses in oxygenation changes that are probably associated with the relatively low climbing level of our participants. Participants with a higher climbing level might show lower interindividual differences due to better habituation to climbing-specific tests.

In summary, our results suggest that wearing forearm compression sleeves over the finger flexor muscles did not enhance hand grip strength and endurance or the sports climbing performance parameters of finger hang, lap climbing distance and time, nor did it affect maximal blood lactate values or the parameters of perceived exertion and muscle pain after lap climbing. However, wearing forearm compression sleeves resulted in slightly higher changes in oxy[heme] and StO_2_ during the deoxygenation and reoxygenation phases of hand grip strength and endurance measurements, but did not alter the total[heme] concentration changes or hemodynamic changes in finger hang measurements. Therefore, we could not confirm any benefit of the use of forearm compression sleeves during climbing exercise for a performance enhancement in sports climbing and bouldering this study, and claims of benefits should be considered with caution.

## Data Availability

The raw data supporting the conclusions of this article will be made available by the authors, without undue reservation.
